# Extracellular Vesicles—New Players in Cell-to-Cell Communication in Gestational Diabetes Mellitus

**DOI:** 10.3390/biomedicines10020462

**Published:** 2022-02-16

**Authors:** Carlos Palma, H. David McIntyre, Carlos Salomon

**Affiliations:** 1 Exosome Biology Laboratory, Centre for Clinical Diagnostics, The University of Queensland, Brisbane, QLD 4029, Australia; c.palma@uq.edu.au; 2 Centre for Clinical Research, Royal Brisbane and Women’s Hospital, The University of Queensland, Brisbane, QLD 4029, Australia; 3 Mater Research, The University of Queensland, Brisbane, QLD 4101, Australia; h.d.mcintyre@uq.edu.au; 4 Departamento de Investigación, Postgrado y Educación Continua (DIPEC), Facultad de Ciencias de la Salud, Universidad del Alba, Santiago 8320000, Chile

**Keywords:** extracellular vesicles, gestational diabetes mellitus, pregnancy complications, insulin sensitivity, glucose tolerance

## Abstract

Research in extracellular vesicles (EVs) has contributed to a better understanding of physiological and pathophysiological conditions. Biologically active cargo, such as miRNAs and proteins, is critical in many different biological processes. In this context, pregnancy is one of the most complex physiological states, which needs a highly regulated system to ensure the correct nourishment and development of the baby. However, pre-existent maternal conditions and habits can modify the EV-cargo and dysregulate the system leading to pregnancy complications, with gestational diabetes mellitus (GDM) being one of the most reported and influential. Calcification and aging of muscle cells, protein modification in vascular control or variations in the levels of specific miRNAs are some of the changes observed or led by EV populations as adaptation to GDM. Interestingly, insulin sensitivity and glucose tolerance changes are not fully understood to date. Nevertheless, the increasing evidence generated has opened new possibilities in the biomarker discovery field but also in the understanding of cellular mechanisms modified and involved in GDM. This brief review aims to discuss some of the findings in GDM and models used for that purpose and their potential roles in the metabolic alterations during pregnancy, with a focus on insulin sensitivity and glucose tolerance.

## 1. Introduction

Hyperglycaemia that develops during pregnancy and resolves after birth (gestational diabetes mellitus (GDM)) has been recognised for over 50 years as a pregnancy complication [1], but different expert panels have been unable to agree on the uniform global diagnostic values for GDM and optimal treatment protocols [2,3]. GDM is one of the most common medical complications of pregnancy, generally detected between 24 and 28 weeks of gestation [4,5]. However, hyperglycaemia may be present and undiagnosed from before pregnancy or from early gestation, related to the increasing prevalence of prediabetes, type 2 diabetes mellitus (T2DM) and obesity in the population. Other factors such as parental overweight and high fat percentage, family history of type 2 diabetes mellitus and ethnicity, among other factors, are also recognised as high-risk factors for the development of GDM. An additional problem is that offspring of GDM mothers carry a high risk of developing transgenerational chronic diseases including, for female offspring, a high risk of developing GDM later in life [6]. In these circumstances, early detection or screening have already shown benefits by allowing proper management of cases, fetal monitoring of potential critical pregnancies and provision of optimal antenatal care for GDM mothers [7,8]. The existence of different factors including unhealthy habits (e.g., smoking, diet, sedentarism), psychosocial factors, in addition to the biological ones already present (e.g., overweight/obesity, age, ethnicity, genetic factors, etc.) that increase the risk of developing GDM is a challenge that needs to be addressed to establish universal biomarker-based screening to identify women early during pregnancy at risk to develop GDM [1].

In the words of the Biomarker Definitions Working Group (2001), a biomarker is understood as “a characteristic that is objectively measured and evaluated as an indicator of normal biological process, pathogenic process or pharmacologic responses to a therapeutic intervention” [9]. In more concrete terms, a biomarker must be a reproducible and rapidly measurable molecule, which provides accurate clinical information necessary and useful for early diagnosis or management of a pathological condition. Recent studies highlight the potential utility of Extracellular Vesicles (EV) in the diagnosis of disease onset and treatment monitoring [10,11,12,13]. 

**Figure 1:**
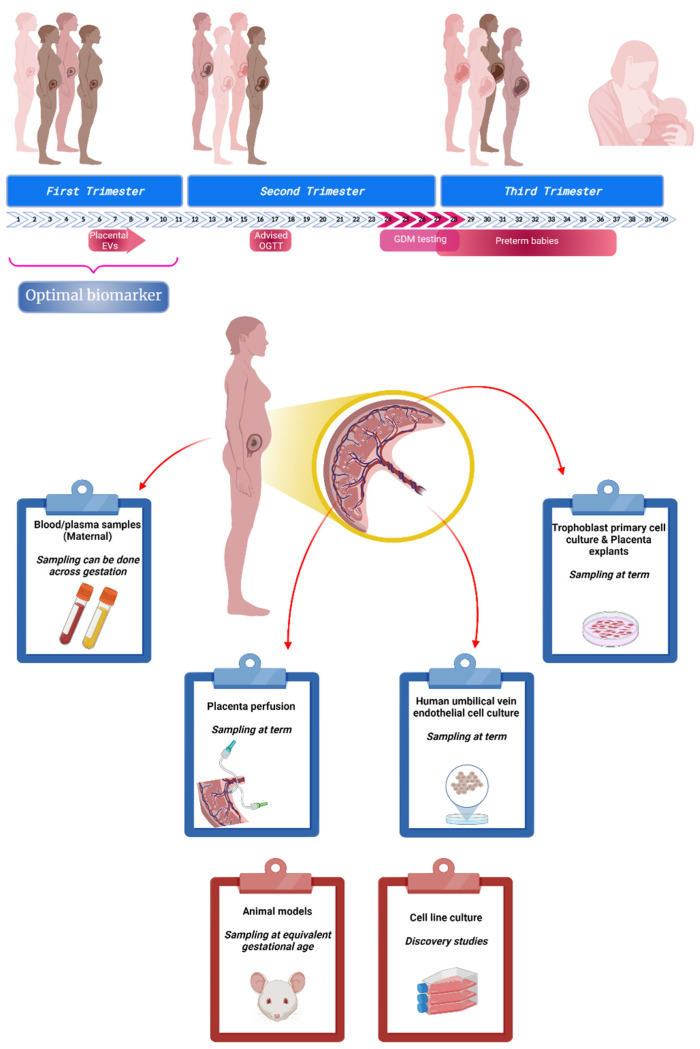
Biomarker discovery in gestational diabetes mellitus (GDM). Pregnancy is a complex physiological state where, in 40 weeks, a sequence of adaptations take place in order to assure the correct nourishment and development of the baby. Currently, GDM affects a large proportion of mothers and its screening test takes place around week 24–28 of gestation. At this point, other pregnancy complications may be present (co-morbidities) putting at risk not just the baby (e.g., preterm birth) but also the mother. New guidelines and non-evidence based recommendations are trying to address this issue, advising new oral glucose tolerance test (OGTT) evaluation early in pregnancy. Nowadays, several research groups around the world are focused on identifying potential biomarkers for GDM in the early stages of pregnancy to support and optimise the resources needed for a successful outcome. This research uses different models and approaches to generate evidence. It can be used plasma samples containing molecules or components with a placental origin; placenta that can be used for perfusion or primary cell culture; or other adjacent structures such as umbilical cord to study interaction and other control points. In addition to these models, animals and immortalized cells have been used to test different conditions/drugs and to get a better understanding of cellular mechanisms involved in these pathological conditions. (Created with BioRender).

To date there is a paucity of data defining changes in the release, role and diagnostic utility of circulating EVs in pregnancies complicated by GDM. The presence of placenta-derived EVs in maternal circulation have been characterised by the co expression of a placental marker (i.e., placental alkaline phosphatase (PLAP)) [12,14], which has allowed detection of changes in the levels and content of EVs in GDM [11,15,16,17,18]. Thus, the aim of this review is to discuss the current criteria of diagnosis of GDM and how EV research might have a potential role in the early identification of women at risk to develop GDM. We propose that circulating EVs during pregnancy might interact with insulin target organs and regulate key processes involved in maternal metabolic adaptation to pregnancy including insulin sensitivity, secretion and glucose tolerance, key elements present in GDM. As the technologies improve and allow the development of more sensitive and specific detection platforms/devices, the use of EVs as a source of biomarkers increases and opens new opportunities in their clinical application for the diagnosis of diseases [19]. An overview of optimal biomarker discovery timeline and experimental models to understand the role of EVs in GDM is presented in Figure 1.

## 2. GDM Overview and Clinical Associations

GDM is one of the most common medical complications of pregnancy worldwide. The International Diabetes Federation estimated 16.4 million live births were complicated by GDM in 2019. This hyperglycaemia condition during pregnancy is related to the development of pregnancy complications including excess fetal growth [20], hypertensive disorders of pregnancy [21] and the later maternal and offspring transgenerational effects including high risks of obesity, hypertension and diabetes. 

As for other diseases or disorders, the etiology of GDM is multifactorial involving a pathophysiological state as well as a genetic component [6]. As aforementioned and also discussed later, there is no consensus on a unique and specific set of guidelines for case detection and management of GDM or monitoring high-risk pregnancies [22]. Conventional GDM diagnosis is usually made between 24–28 weeks of gestation, but in many cases, the glucose levels may have been elevated from early pregnancy or before conception, given the high rates of diagnosed and undiagnosed prediabetes and diabetes in many populations. However, evidence suggesting that early diagnosis of GDM allows early intervention and might be associated with a reduction in large-for-gestational-age (LGA) infants is unclear. For instance, GDM cases detected before 24 weeks of gestation presented a higher BMI and lower gestational weight gain compared with those diagnosed after 24 weeks, even though neonatal complications, such as neonatal intensive care unit admission or small-for-gestational-age, were not observed [7]. Previously, it was shown in Australia that macrosomia, LGA and neonatal intensive care admission cases were comparable between GDM patients and pre-existing diabetes patients, even if the diagnosis of GDM was before 12 weeks of gestation [23]. Another study in Morocco revealed how the inclusion of early screening for diabetes allows improvements to the algorithm used for detection and initial management. At the same time, this prompt identification of GDM cases allows the contribution of different medical specialists to monitor fetal development and the mother’s health throughout the pregnancy [8]. Thus, if early detection tests were available, they would represent a major advance and contribution to the discipline and afford the opportunity to evaluate alternate treatment and clinical management strategies to improve health outcomes for both mother and baby. However, the lack of a universal gold standard for GDM screening has led to diverse algorithms and criteria for the identification of GDM, thus, limiting accurate assessment of the prevalence of GDM [3].

## 3. Inconsistencies in the Diagnostic Assessment

Around the world, several evaluation protocols have been developed for the screening and detection of GDM. Nowadays, there are a few suggestions that are not evidence-based for testing and management of dysglycaemia. Values between 5.1 and 5.9 mM for fasting plasma glucose level (FPG) indicate significant uncertainty, and it is advised to have an oral glucose tolerance test (OGTT) at 16–18 weeks. However, values over 6.0 mM (until 6.9 mM) indicate a potential later diagnosis of GDM. Evaluation of random plasma glucose is not clear even when the level is high. In the case of glycated haemoglobin (HbA1c), values between 5.9 and 6.7% have shown to have low sensitivity but relatively high specificity for the diagnosis of GDM. However, these studies are not yet conclusive and provide only low-quality evidence. Therefore, more evidence is needed in order to consider HbA1c as a useful diagnostic tool in pregnancy. It may be useful in high-risk populations to record glucose levels and HbA1c as part of a national diabetes services scheme [24,25,26].

As already mentioned, the limits for fasting plasma glucose (FPG) considered for GDM diagnosis are unclear and, in addition, these values have been adjusted several times according to emerging new evidence. These limits are quite similar but need to be considered by the medical team as part of a larger patient background. Currently, 5.1 mM (92 mg/dL) is a common value for FPG levels, at least in the main guidelines for diagnosis and management of pregnancy (Table 1). 

In the study of GDM and improvements for the diagnosis protocol, an important contribution was made in 2008 when the results of the Hyperglycemia and Adverse Pregnancy Outcome (HAPO) study were published. This international multicentre cohort study consisted of 25,505 pregnant women, who were tested with a blinded 2-h 75 g OGTT between 24 and 32 weeks’ gestation and then followed (without treatment) throughout pregnancy to detect primary and secondary outcomes (HAPO study). The study demonstrated a continuous association between plasma glucose levels and adverse pregnancy outcomes. The analysis was performade after adjusting multiple potential confounders and these associations were independent of other known risk factors for these outcomes. As a result of the HAPO study and other studies in which were associated glycaemia levels of the mother and perinatal/long term outcomes, the International Association of Diabetes and Pregnancy Study Groups (IADPSG) consensus panel generated the most widely used guidelines for the diagnosis of GDM [27]. These guidelines were later endorsed by the FIGO Initiative (group of countries that represents 55% of the global live births and 55% of the global burden of diabetes) [28] and another expert panel, the American Diabetes Association. The screening protocol recommended diagnostic thresholds using a fasting 75 g OGTT of: FPG 5.1 mM (92 mg/dL), 1 h 10.0 mM (180 mg/dL) and 2 h 8.5 mM (153 mg/dL) [27,29].

**Table 1:** Summary of main guidelines for gestational diabetes mellitus screening and diagnosis.

Guideline	Fasting Plasma Glucose (FPG) Cut-Off	Oral Glucose Tolerance Test (OGTT) (or Glucose Challenge Test (GCT))	1-h Threshold	2-h Threshold	Observation	Ref.
International Association of Diabetes and Pregnancy Study Groups (IADPSG)	5.1 mM (92 mg/dL)	75 g	10.0 mM (180 mg/dL)	8.5 mM (153 mg/dL)		[30]
American Diabetic Association (ADA)	5.1 mM (92 mg/dL)	75 g	10.0 mM (180 mg/dL)	8.5 mM (153 mg/dL)		[29,31]
	5.3 mM (95 mg/dL)	50 g	10.0 mM (180 mg/dL)	8.6 mM (155 mg/dL)	3 h: 7.8 mM (140 mg/dL) At least two measures above limit.	
Royal Australian and New Zealand College of Obstetricians and Gynaecologists (RANZCOG)	5.1 mM (92 mg/dL)	75 g	10.0 mM (180 mg/dL)	8.5 mM (153 mg/dL)	Initial 1-h non-fasting oral Glucose Challenge Test (GCT) is no longer recommended	[32]
New Zealand Society for the Study of Diabetes (NZSSD)	5.5 mM (99 mg/dL)	75 g	-	9.0 mM (162 mg/dL)	Glycated haemoglobin (HbA1c) (week 20)	[33]
Royal Australian College of General Practitioners (RACGP)	5.5 mM (99 mg/dL)	75 g	-	8.0 mM (144 mg/dL)		[34,35]
Australasian Diabetes In Pregnancy Society (ADIPS)	5.1–6.9 mM (92–125 mg/dL)	75 g	10.0 mM (180 mg/dL)	8.5–11.0 mM (153–199 mg/dL)	Suggested an early OGTT (or HbA1c) with first antenatal blood or at the first antenatal visit (in the first trimester), register of glucose levels (National diabetes services scheme)	
Canadian Diabetes Association (CDA)	5.3 mM (95 mg/dL)	50 g	10.6 mM (190 mg/dL)	9.0 mM (162 mg/dL)	If abnormal, 75 g OGTT	[36]
	5.1 mM (92 mg/dL)	75 g	10.0 mM (180 mg/dL)	8.5 mM (153 mg/dL)		
World Health Organization (WHO)	5.1–6.9 mM (92–125 mg/dL)	75 g	10.0 mM (180 mg/dL)	8.5–11.0 mM (153–199 mg/dL)	No established criteria for the diagnosis of diabetes based on the 1 h post-load value	[37]

The challenge represented by the diagnosis of GDM is in part explained by the existence of several factors that can affect the values of FPG or the condition of the patient. Evidence has related ethnicity and BMI as important funders in the development of GDM but also revealed how these differences have an important repercussion in the strategies used for better management of the patient [38]. Recommendations of the National Institute for Clinical Excellence (NICE—UK) to follow in the booking appointment are Body mass index (BMI) above 30 kg/m^2^, previous macrosomic baby (>4.5 kg), previous GDM, history of a first-degree relative with diabetes, and ethnicity [39]. These recommendations are used especially when testing is not universal, but rather selective for determining which patient has a higher risk of developing GDM as a result of the interaction between ethnicity, lifestyle, preexistent conditions, among many other risk factors; all of them not considered in the algorithm [35,39,40].

Current research in identifying biomarkers that may be detected earlier in pregnancy (e.g., <20 weeks) is challenging due to the uncertainty and variation in diagnostic criteria for GDM. Recently, evidence supporting a role for EVs in cell-to-cell communication during pregnancy, and especially in GDM, has been obtained. EVs are secreted by the placenta during pregnancy and their release may correlate with pregnancy outcome [40,41]. (REF). in the next sections, we discuss the potential role of EVs in GDM, and the experimental models used to identify EV-associated pathways in GDM. 

## 4. Extracellular Vesicles: New Players in Cell-to-Cell Communication

“Extracellular vesicles” is an umbrella term that groups diverse membranous components that can be found in biological fluids. The main separation criteria used in research have been based on their physical features such as size or protein marker expression. The differential expression of proteins on the surface of these vesicles has allowed the profiling and separation according to the originating cell type or tissue and consequently, the development of monitoring tools. Due to the mixed origin of EV preparations and the absence of specific EV subtype markers, the International Society of Extracellular Vesicles has recommended the use of size to categorise EVs [42]. Following these criteria, it is possible to distinguish 2 main groups: small EVs (sEVs—size < 100–200 nm) and medium/large EVs (m/lEVs—size > 200 nm). Another physical characteristic is density, which usually classifies them in low, medium and high density. However, the development of a classification method based on the use of these physical features is insufficient since there is an overlap between different extracellular vesicles or lipids in circulation [42]. 

In order to have a better understanding of the different vesicle populations, classifications have also included biochemical composition and descriptions of conditions or originating cells to clearly identify the specific type of vesicle under study (Table 2). In a biochemical context, the expression of tetraspanins, ligands or phospholipids, among others, can help in the categorisation of extracellular vesicles in sEVs, ectosomes (or microvesicles) and apoptotic bodies. In terms of the source of these EVs, we can identify for example placenta-derived EVs, hyperglycaemic EVs, large oncosomes and apoptotic bodies [42]. 

**Table 2:** Summary of extracellular vesicle features.

Vesicle	Size	Biogenesis	Main Markers	Density	Content	Ref.
Small Extracellular Vesicles (sEVs)—previously identified as exosomes	30–100 nm	Budding of the cellular plasma membrane and later inward invaginations of this endosomal membrane	ESCRT machinery (TSG101, Alix, HRS), Tetraspanins (e.g., CD9, CD63), RABs	1.08–1.19 g/mL	Proteins, mRNA, miRNA, lipids	[43,44,45]
Ectosomes (microvesicles or microparticles *)	>100–1000 nm	Direct plasma membrane fission	Tubulin, CD40, Integrins, selectins	~1.15 g/mL	Proteins, mRNA, miRNA, lipids	[43,46,47,48,49,50]
Apoptotic bodies	500–5000 nm	Programmed cell death process	Annexin V, phosphatidylserine	1.16–1.28 g/mL	Organelles, proteins, DNA, different RNA species, lipids	[51,52,53,54]

* Discouraged term because of potential misinterpretation related to synthetic nanomaterials.

In terms of content (cargo) of EVs, evidence generated in sEVs suggests a regulated mechanism of packaging for several proteins and other nucleic acid species [55], although the details of the molecular mechanism of packaging remain unclear. This cargo includes not only markers such as ESCRT components or integrins, but a wide range of molecules. Evidence indicates that sEVs contain proteins such as cytoskeletal proteins, enzymes, signal transducers, heatshock proteins, as well as nucleic acid species such as mRNA and miRNAs and also CircRNA, piRNA, Y-RNA and viral DNA. Aminoacids and different lipids have been also reported as cargo [56,57,58,59,60,61]. Microvesicles, on the other hand, have also been shown to transfer proteins, miRNA and mRNA mutant/variants [62,63,64,65] (Table 2). Due to the nature of apoptotic bodies, the cargo can include proteins, RNA and lipids but in addition, organelles, fragments of DNA and other components produced during the cell-death process [51]. This cargo may be appointed by the originating cell [66] as a response that requires specific bioactivity to tissue physiology conditions such as glucose bioavailability, reactive oxygen species, oxygen tension, free fatty acid concentration, among others [67,68,69]. In fact, in vitro experiments using first-trimester trophoblast cells have demonstrated that the release rate, as well as the cargo and bioactivity of sEVs, can be modified by exposure of originating cells to different oxygen tension [67]. In addition, it has been reported that sEVs isolated from a trophoblast cell line exposed to high glucose concentration (mimicking GDM conditions) [69] can promote processes such as cell migration and apoptosis in a different cell type [70]. Thus, EV biology is a promising field of research, and current data strongly suggest that EV signaling is involved in the regulation of glucose homeostasis during gestation and may contribute to the development of GDM, through alterations in bioactive molecules including proteins and nucleic acids. 

## 5. Extracellular Vesicle-Associated Changes in the Pathophysiology of GDM

The average concentration of FPG is 90 mg/dL (5.0 mM) in nonpregnant women. In pregnancy, this value decreases across gestation [71]. In addition, pregnancy is associated with an increase in glucose release by the liver with a concomitant increase in the concentration of insulin [72,73]. At the same time, there is a decrease in hepatic insulin sensitivity which is further decreased in pregnant obese patients [74]. In the case of GDM, the FPG is higher but without an increase in the glucose release from the liver. Insulin levels in these patients are higher compared to pregnancies without GDM [72]. EVs have been associated with changes in glucose intolerance [75]. EVs transfer miR-99b from adipose tissue to the liver that partially inhibits the expression of hepatic fibroblast growth factor 21 (FGF21) mRNA improving insulin sensitivity [75]. Changes in insulin sensitivity have also been observed in adipocytes, myocytes and hepatocytes as a result of overexpressed EV miR-29a derived from adipocyte tissue macrophages in obese mice [76]. Conversely, it has been demonstrated that EVs released by adipose tissue macrophages from obese mice exhibit an upregulation of miR-155 which is related to glucose intolerance and insulin resistance, an effect reverted in miR-155KO. When the EVs used to treat obese mice are generated in adipose tissue macrophages from lean mice, an improvement in insulin sensitivity is observed. In addition to these findings, the use of Rosiglitazone (a PPAR-γ agonist) or Cardarine (a PPAR-δ agonist) improves glucose tolerance and insulin sensitivity when tested in 3T3-L1 adipocytes, L6 muscle cells and primary hepatocytes [76,77]. Conversely, the repression of PPAR-γ by adipocyte-derived EV miR-27a and the subsequent decrease in expression of IRS-1 and GLUT-4 has been shown to correlate with obesity and insulin resistance when analysed in C2C12 skeletal muscle cells [78]. Interestingly, miR-27a is one of the miRNAs identified as downregulated in placental tissue of GDM patients [79].

There is an increasing demand for insulin across gestation. Thus, the function of pancreatic β cells is critical in appropriately increasing insulin release in response to reduced peripheral insulin sensitivity. It has been shown that, in cases of T2DM, the pancreatic β cells release EVs containing lower levels of miR-26a. The evidence generated indicates that miR-26a in β cells improves insulin resistance and hyperinsulinemia resulting from obesity [80]. Related to GDM, it has been evidenced that this miRNA is elevated in maternal peripheral blood of GDM patients compared to normal pregnancies in patients 3 to 11 years after delivery. It is important to mention that this miRNA was part of a panel of 28 other miRNAs evaluated for the study of the association of the previous GDM and GDM and Cardiovascular and Cerebrovascular Diseases [81].

EVs can also carry proteins that can lead to changes in insulin sensitivity. EVs secreted from adipose tissue obtained from obese mice can induce macrophage activation through TLR4/TRIF pathway by the action and overexpression of retinol-binding protein 4 (RBP4) impairing glucose uptake and the insulin response of myocytes evaluated in vitro [82]. Interestingly, the expression of circulating RBP4 was evaluated in pregnant women (between 4–10 weeks) and the results evidenced a strong association between RBP4 levels and the risk of developing GDM [83]. RBP4 was also suggested as the main mediator in the relation between Pittsburgh Sleep Quality Index (PSQI) and Insulin resistance in early pregnancy [84].

Levels of Sonic Hedgehog (Shh) within circulating EVs are higher in patients with type 2 diabetes (DT2) compared to controls without DT2. Interestingly, EVs are released in response to high glucose and insulin from adipocytes and induced pro-inflammatory state of bone marrow-derived macrophages (BMDM) and RAW 264.7 macrophages. This mechanism seems to be mediated through Ptch/PI3K signalling pathway and contributes to insulin resistance in adipocytes [85]. Bioinformatic analysis and posterior validation with Western blot and real-time qPCR of human umbilical vein endothelial cells (HUVECs) obtained from GDM patients showed that Shh and the transcription factor E2F1 were significantly downregulated in cases of GDM compared with non-GDM; whereas Homeobox A9 (HOXA9) and Signal Transducer And Activator Of Transcription 1 (STAT1) were upregulated [86]. Absence of Sirtuin-1 (SIRT1) in adipocytes in vivo, a key sensor and regulator of lipid metabolism, increases the body weight and fat mass in mice. SIRT1-deficient mice demonstrate increased release of EVs from adipose tissue with a potential systemic effect on metabolic regulation [87] leading to impaired glucose tolerance and insulin sensitivity. It has been determined that GDM patients show differences in the expression of other proteins such as spectrin alpha erythrocytic (SPTA)-1, CAMK 2β, PAPP-A, Perilipin 4, fatty acid-binding protein (FABP) 4 and hexokinase-3 [17], and all proteins were previously shown to also be differentially expressed and related to insulin resistance [88,89,90]. Taken globally, these data suggest that EVs are involved in changes in insulin sensitivity by transferring bioactive molecules to target cells that might lead to GDM. 

## 6. Extracellular Vesicle-Associated Signaling in GDM

Circulating placental and non-placental EVs during GDM pregnancies may also alter maternal physiology via transfer of miRNAs, proteins, and other bioactive molecules that have been specifically preconditioned by the GDM environment. These may influence maternal response systems. It has been shown that first-trimester primary trophoblast cells exposed to a high concentration of D-glucose (25 mM) and under different oxygen tensions significantly increased their release of EVs from trophoblast cells [69]. Analysis of EVs present in maternal circulation indicates that the concentration of total circulating EVs increases in the plasma of pregnant women compared to non-pregnant women, however, the total number of EVs evaluated in the circulation of women with GDM has shown to be higher compared to controls, and it can be observed across gestation. Additionally, the same pattern is observed when the placental fraction (EV-PLAP^+^) is evaluated [11]. 

In terms of function, it has been shown that EVs from GDM patients can significantly increase the release of proinflammatory cytokines from human umbilical vein endothelial cells (HUVECs) [11], which can contribute and exacerbate the already present proinflammatory state. The effect of these sEVs can be even higher in cases where, in addition to GDM, the EVs were derived from obese women (as compared to those who were lean and overweight) [12]. EVs released by HUVECs significantly increased when these cells are exposed to high glucose concentrations. Interestingly, EVs obtained from cells exposed to high glucose also showed increased endothelial cell wound-healing compared with EVs from HUVECs cultured under basal glucose concentration. This study demonstrated that the hyperglycemic mimetic condition increased the expression of P~Ser^1177^-eNOS, hCAT-1 and VEGF. 

In addition, EVs obtained in high glucose exposure also increased P~Ser^1177^-eNOS. In terms of mRNAs, eNOS and hCAT-1 are increased after high glucose treatment. EVs from a diabetic environment can increase VEGF mRNA. In this model, high glucose increased the expression of ICAM-1 in HUVECs [91]. Evidence also indicates that EVs from HUVECs exposed to 30 mM of glucose induce the calcification and aging of human vascular smooth muscle cells. The findings showed enrichment of Notch3 and determined that calcification and aging processes are enhanced through the mTOR signaling pathway [92].

Dipeptidyl peptidase IV (DPPIV) is associated with changes in insulin sensitivity by breaking down glucagon-like peptide-1 (GLP-1), which in turn regulates glucose-dependent insulin secretion. Interestingly, levels of EV-DPPIV are higher in GDM compared to normal placentae using a placental perfusion system [93]. Analysis of gingival crevicular fluid at 11–14 weeks as the source of EVs revealed that the total number of EVs in this matrix is also higher in GDM patients compared to controls [94]. Conversely, a different fraction related to EVs released by adipocytes has been observed to decrease in maternal circulation in cases of GDM [95]. 

Recently, it has been identified that miRNAs, miR-16-2-3p, miR-16-5p, miR-1910-5p, miR-423-5p, miR-92a-3p and miR-92b-3p are differentially expressed in GDM compared to normal glucose tolerance (NGT) pregnancies [17]. Interestingly, the expression of miR-423-5p and miR-16-2-3p is reported to be similarly altered in type 2 diabetes [96,97]. The expression of a selected set of miRNAs in placenta, plasma and skeletal muscle biopsies from NGT and GDM has been evaluated. Interestingly, the expression of miRNAs varied in a consistent pattern in the placenta, in circulating EV, and in skeletal muscle in GDM [18]. These data suggest that circulating EVs during GDM pregnancies carry biologically active molecules that have the potential to regulate maternal insulin sensitivity.

## 7. Conclusions and Future Directions

GDM is one of the most common complications of pregnancy and represents for mother and baby a high risk for developing transgenerational chronic diseases, including diabetes and cardiovascular disease. GDM, along with other pregnancy complications and poor lifestyle quality, has no single origin but multiple factors and maternal phenotypic features have been identified as contributors to the establishment of this pathological state. 

Studies to dates have focused on the difficult challenge of identifying a universal biomarker, which is perhaps unlikely given the diverse nature of GDM. Further, the variable diagnostic criteria assessment currently employed for detection of GDM is another challenge faced by the medical community and researchers. These factors may explain why a universal GDM biomarker remains elusive. 

Nevertheless, research in the field of EVs is generating valuable evidence for a better understanding of the alterations observed in cell-to-cell communication in GDM pregnancies. EVs can be detected in all body fluids studied until now, including maternal blood, and contain bioactive molecules such as proteins and nucleic acids, representative of the cell of origin. Thus, EVs contain cellular markers from difficult-to-access anatomical sites, making them strong candidates as biomarkers of disease. 

Current evidence suggests that EVs are involved in maternal glucose homeostasis in GDM, by regulating insulin signaling in skeletal muscle and insulin secretion from pancreatic b cells. Although the mechanism(s) associated with the effect of EVs in GDM are not fully established, circulating EVs can transfer their contents to other cells, a process that is important to several biological processes including insulin sensitivity and glucose homeostasis. 

**Figure 2:**
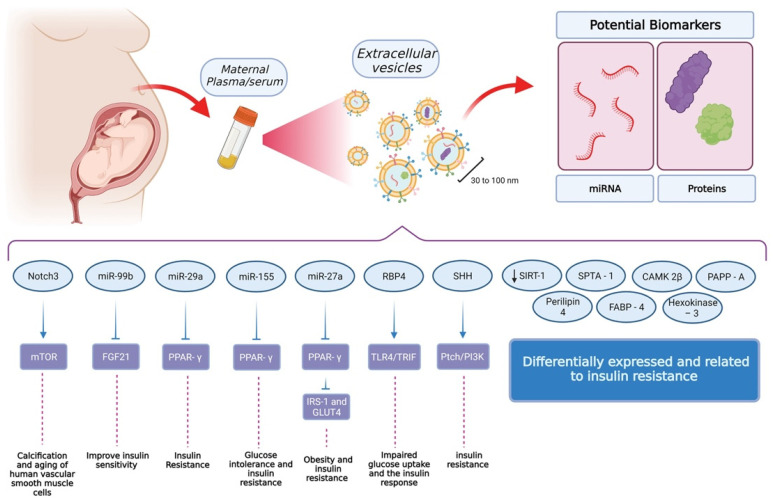
Findings related to extracellular vesicles and their role in insulin sensitivity and glucose tolerance. The standard procedure for the isolation of extracellular vesicles uses maternal plasma or serum as the source. From these samples, it is possible to isolate vesicles with a size between 30 and 100 nm. Interestingly, these vesicles contain miRNA and proteins as cargo, which have shown to be attractive options in the field of biomarker discovery. So far, these cargoes have been identified as critical elements for insulin resistance and glucose tolerance but also, at a higher level, processes involving muscular and adipose tissue. Other elements have been identified but further research is needed in order to reveal their function and relevance at a cellular level. (Created with BioRender).

Nowadays there is a better comprehension of how transient tissues only observed during pregnancy respond to a maternal physiological state and modify the information contained as cargo of EVs to orchestrate other functions (Figure 2). From a GDM point of view, there is increasing evidence related to the effect of the plasma glucose level in EV-cargo and its role in insulin sensitivity and glucose tolerance. Currently, the mechanisms involved in the sensing process by the placenta and other tissues, as well as the cellular mechanisms employed by the cell to sort different cargoes, are not well understood. 

Future challenges in the EV field include the standardisation of methods to isolate or enrich EVs, understanding the role of different subtypes of EVs (i.e., heterogeneity), and the identification of their targets (i.e., in vivo biodistribution). In the biomarker field, concerns about the reproducibility of the isolation methods and quantification of the molecules within EVs are critical parameters that need be addressed. If reproducibility and high sensitivity and specificity can be achieved, EVs may prove to be valuable biomarkers for early assessment of risk and the application of an appropriate clinical management strategy to improve outcome for GDM pregnancies.

## Data Availability

Not applicable.
